# Destroying Nerves of the Human Teeth

**Published:** 1847-10

**Authors:** J. Allen


### DR. J. ALLEN, *On destroying the Nerves of the Human Teeth.*

The practice of destroying the lining membranes of teeth, after they have been exposed to the action of external agents, in consequence of decay, has become very common. The writer therefore deems it proper to report the result of his experience and observations upon that subject. In the adoption of any theory or mode of practice, either in the medical or dental profession, it is important that sound principles well attested, should be the foundation upon which they are based: Let us then examine the subject of killing the nerves of decayed teeth, and see whether it be a means of lessening or a means of increasing the aggregate amount of human suffering. A. had a tooth decayed to the lining membrane, and was very anxious to have it saved. The nerve was destroyed, and the tooth well filled, after which it ceased to be troublesome, and was again useful. Five years have elapsed, and that tooth is still rendering essential service, and has given little or no trouble since it was filled. B. had a tooth, the nerve of which was exposed and had been exceedingly troublesome for a long time. He also had the nerve killed, and the tooth pluged; but in about a week it became very painful: the filling was removed—the pain ceased, four days after it was refilled, but soon it became painful again; on removing the second plug, the pain subsided – it then remained without filling three or four months. It was then again refilled, and soon after became very sore and troublesome, and was removed. C. D. E. & F. had also teeth affected in a manner similar to those above mentioned, and with similar results. G. and H. had each a nerve killed: the teeth were filled, and thereby rendered useful for several months, but at length became sore from the effect of cold—ulcerations ensued at the apex of the fangs, and were necessarily removed. From the foregoing we find that one tooth out of the six thus treated was rendered useful and valuable; two others were temporarily restored, but finally lost; the other three were total failures—protracting suffering under the delusive hope of realizing a perfect restoration of those diseased teeth. These cases exhibit an average proportion of the number of teeth that may be rendered useful by filling after decay has extended to the lining membrane. It may be asked, if one tooth can be saved by filling after decay has reached the nerve, why may not another under the same apparent favorable circumstances be confidently expected to realize a like result? If the nerve of a tooth has been exposed but a short time, and the investing membrane yet unaffected by it, in a healthy mouth the nerve may be killed and then thoroughly removed; which can be done with small elastic instruments, with sharp retangular points for cutting off and drawing out the nerve from near the apex of the fang. This done the tooth may be filled and rendered useful for many years. But if the lining membrane of a tooth has become exposed in an unhealthy mouth, and the investing membrane also become affected, then a successful result should not be anticipated; and unfortunately a very large proportion of the cases we are called upon to treat in this manner are of this class. Consequently the indiscriminate practice of destroying nerves and then filling the teeth must be fraught with consequences prejudicial to the profession, and deleterious to the health and comfort of the patients, and is at war with sound pathological principles.

